# Editorial: High-level antimicrobial resistance or hypervirulence in emerging and re-emerging “super-bug” foodborne pathogens: detection, mechanism, and dissemination from omics insights

**DOI:** 10.3389/fmicb.2024.1459601

**Published:** 2024-08-09

**Authors:** Yujie Hu, Wei Wang, Scott Van Nguyen, Guerrino Macori, Fengqin Li, Séamus Fanning

**Affiliations:** ^1^NHC Key Laboratory of Food Safety Risk Assessment, Chinese Academy of Medical Science Research Unit, China National Center for Food Safety Risk Assessment, Beijing, China; ^2^Sequencing and Bioinformatics Center, American Type Culture Collection, Washington, DC, United States; ^3^School of Biology and Environmental Science, University College Dublin, Belfield, Ireland; ^4^UCD-Centre for Food Safety, School of Public Health, Physiotherapy and Sports Science, University College Dublin, Belfield, Ireland; ^5^Institute for Global Food Security, School of Biological Sciences, Queen's University Belfast, Belfast, United Kingdom

**Keywords:** antimicrobial resistance (AMR), hypervirulence, foodborne pathogen, mechanism, dissemination, omics

With the development of the global livestock and poultry industry, food processing industry and international trade, the spread of foodborne pathogenic microorganisms is accelerating and becoming more complex. This has resulted in a variety of foodborne diseases and increasingly serious antimicrobial resistance (AMR), which have brought an incremental disease burden to the health of people around the world. This is already one of the most challenging public health issues internationally and still causes a substantial economic and social burden worldwide (Pires et al., 2021).

The most common pathogens responsible for spreading foodborne diseases in humans include but are not limited to *Salmonella, Campylobacter, Clostridium, Cronobacter*, pathogenic *Escherichia coli* (*E. coli*), *Listeria monocytogene* (*L. monocytogene*), *Staphylococcus aureus* (*S. cureus*), *Vibrio parahaemolyticus* (*V. parahaemolyticus*), *Bacillus cereus* (*B. cereus*), *Yersinia enterocolitica* (*Y. enterocolitica*), among others. These pathogens contaminate various types of foods throughout the food chain including cereal, vegetables, fruits, meat, dairy, and aquatic products in entire proceedings from farmland to fork and disseminate AMR and virulence.

In addition to the virulence-related phenotypes expressed, different metabolites, including various toxins that can be produced, several pathogenic bacteria of the so called “ESKAPEE” group, that often express an AMR phenotype, can cause serious human illnesses, resulting in a substantial disease burden. According to estimates published in 2019, 1.27 million deaths were directly attributed to drug-resistant infections globally, and 4.95 million deaths were associated with bacterial AMR in total (Murray et al., 2022). Some estimates also indicate that by 2050 there could be up to 10 million deaths globally per year—on par with the 2020 worldwide death toll from cancer (O'Neill, 2016; United Nations Environment Programme, 2023).

The term “ESKAPE” is composed of seven different pathogens with continuously expanding multidrug resistance (MDR) and virulence phenotypes, responsible for majority of nosocomial infections and are capable of “escaping” the biocidal action of antimicrobial agents (Mulani et al., 2019) the World Health Organization (WHO) published a list of pathogens for which new antimicrobial compound development was urgently needed in an effort to focus and guide research and development related to new antibiotics (Tacconelli et al., 2018; De Oliveira et al., 2020). Within this broad list, ESKAPEE pathogens were designated “priority status”, e.g., Carbapenem resistant *A. baumannii* and *P. aeruginosa* (CRAB and CRPA) along with extended spectrum β-lactamase (ESBL) or carbapenem resistant *K. pneumoniae* (CRKP) and *Enterobacter* spp. are listed in the critical priority list of pathogens; whereas, vancomycin resistant *E. faecium* (VRE) and methicillin and vancomycin resistant *S. aureus* (MRSA and VRSA) are in the list of high priority group (Mulani et al., 2019). Building on the 2017 edition, WHO released a Bacterial Priority Pathogens List (BPPL) in May 2024, updating and refining the prioritization of 15 families of antimicrobial resistant bacterial pathogens and grouping them into critical, high and medium categories for prioritization, to address the evolving challenges of AMR (WHO, 2024).

In this context, these clinically important antimicrobial-resistant foodborne pathogens with high-level AMR or hypervirulence have spread so quickly that they could be found emerging in clinical hospitals, agricultural farmlands, foods, food animals, environments and also humans/animals guts, by virtue of the fact that they have several key biological characteristics, including adaptations for survival in the modern health-care setting, diverse methods for acquiring resistance determinants and the dissemination of successful high-risk clones around the world (Miller and Arias, 2024). However, it is still unclear and understudied as to how prevalent these pathogens are, where they originated from, mechanisms for their extensive resistance and/or hypervirulence, their dissemination, evolution, and potential impacts, and so on. We need more intensive and compelling evidence, explanation, and interpretation. Multi-omics approaches, including genomics, transcriptomics, proteomics and metabolomics etc, are important tools to untangle the genetic, immunologic, (post)transcriptional, (post)translational, and metabolic mechanisms underlying progression from contamination to dissemination, from AMR to pathogenesis, from infection to clearance of the mechanisms of foodborne pathogens (Khan et al., 2019).

This Research Topic aims to provide a platform for recent discoveries and the latest progress in detection, mechanism, and dissemination from omics insights with regards to the emerging or re-emerging foodborne pathogens with high-level resistance or hypervirulence, to increase our understanding of these superbugs, to track their sources, to discover the mechanisms that make them behave thus, and to uncover the dissemination along the animal-food-human chain using big data analysis, to assess human health risks.

*Salmonella* is one of the most common foodborne pathogens causing sporadic cases or outbreaks of gastroenteritis around the world. Salmonellosis, including gastroenteritis and typhoid fever, is the disease mainly caused by consuming food contaminated with *Salmonella*, has a high health and economic burden globally. Poultry meat production supply chain has frequently been associated with human salmonellosis cases and is an important cause of *Salmonella* transmission between poultry farms and humans (Rincón-Gamboa et al., 2021). *Salmonella* is also a significant repository of AMR genes (ARGs), posing substantial challenges to public health and security (Jajere, 2019). Several papers included in this Research Topic reported on AMR and virulence factors: Wang et al. investigated the prevalence, AMR, and genomic characteristics of *Salmonella* isolated from chilled chicken carcasses and humans with diarrhea in Qingdao, China. The most common serotypes (Enteritidis and Typhimurium) and the top four detected resistance phenotypes (to nalidixic acid, ampicillin, tetracycline and chloramphenicol) in *Salmonella* were reported from both chicken and human sources. High prevalence of plasmid replicons and prophages were observed among the isolates and a total of 79 ARGs were found, including ESBL genes, *bla*_NDM − 1_, *mcr-1.1*, and *mcr-9.1*, mediating resistance to these critically important compounds for the clinical treatment of infections caused by ESKAPEE pathogens. Zhou L. et al. uncovered a marked increasing trend in MDR *Salmonella* isolated from retail foods in Guizhou province, China, (2016–2021), with strains from meat products showing significantly higher drug resistance than those from other sources. Their data also showed that *S*. Typhimurium and *S*. Enteritidis were the most prevalent serovars, with a notable presence of MDR strains and key virulence genes. Mora et al. investigated the antimicrobial profiles, virulence, and susceptibility of 105 *S*. *enterica* isolates from swine and chicken samples obtained from slaughterhouses and public wet markets in Metropolitan Manila using whole genome sequencing (WGS) analysis. This study provides proof of principle that WGS approaches can untangle the complex AMR and virulence patterns and shows that sequencing should be implemented by meat inspection authorities to augment the existing presence–absence detection tests as acceptability, safety, and quality criteria. In addition, WGS can also give insights into strain clustering and evidence of infection cross-contamination. Ghoshal et al. analyzed the phenotypic and transcriptomic alterations in the acid-evolved lineages (EL) of *Salmonella enterica* serovar Enteritidis after acid stress exposure to delve into the molecular mechanisms driving adaptive laboratory evolution (ALE) of *Salmonella* in acid stress. They found the elevated antibiotic minimum inhibitory concentration (MIC) observed after exposure to acetic acid for 70 days was lost when acid stress was removed and the MIC swiftly elevated again after stress reintroduction. This phenomenon was observed against several human antimicrobials such as meropenem, ciprofloxacin, gentamicin, and streptomycin. Transcriptomic analysis demonstrated the upregulation of drug resistance, virulence, iron metabolism, and stress adaptation genes during continuous acid stress. The reversible nature of antimicrobial resistance highlights the adaptability of bacterial populations and the potential consequences of stress-induced adaptations. This research provides important insights into the process of adaptive evolution in bacterial populations and underscores the intricate relationship between stress adaptation, antibiotic resistance, and bacterial fitness. This study also emphasizes the importance of the need for a comprehensive and holistic approach, e.g., a combination of genomics and transcriptomics, to understand bacterial responses to stress and their impact on public health and food safety.

Carbapenem-resistant Enterobacterales (CRE), especially *E. coli*, carrying New Delhi Metallo-β-lactamase (NDM) have increased rapidly over the last decades and have become an urgent public health threat (El-Gamal et al., 2017), leading to the increased clinical use of colistin, which has been considered as the last therapeutic option for treating infections caused by MDR microorganisms. However, the efficacy of colistin has been challenged by the emergence of plasmid-mediated mobile colistin resistance (MCR), which was also found in Enterobacteriaceae in 2015 (Liu et al., 2016). Liu et al. reported the first case of *E. coli* carrying *bla*_NDM − 5_ of retail eggs in Guangdong province of China. The IncI1-plasmid-carrying *bla*_NDM − 5_ displayed high homology with a clinical plasmid pEC6363-NDM5, while the IncHI2 plasmid harboring *bla*_NDM − 5_ shared highly similar structures with plasmids of animal origin. Considering the clinical importance of carbapenem together with the fact that the consumption of eggs is substantial in human diet, the carbapenem resistance in eggs has the risk to spread to humans throughout the food chain. Feng et al. identified a colistin resistant *E. coli* ST744 isolate from a fecal sample collected in Shanghai, carrying a conjugable IncI2 plasmid with a stable transferable *mcr-1.1* gene and exhibiting extensive AMR profiles and additional AMR genes, indicating a high risk to disseminate the extensively-drug-resistance phenotype among Enterobacteriaceae.

*S. aureus* exhibits vital adaptability across diverse habitats including food products, human skin, wastewater, treatment plants, public spaces, and households, etc. (Fang et al., 2024). It is also a major cause of infections in both inpatient and outpatient settings. The emergence of MRSA, particularly in communities in the 1990s, raised alarms and resulted in both greater surveillance of MRSA and implementation of strategies to limit MRSA transmission in clinical settings (Carrel et al., 2024). Zhang et al. investigated the prevalence, AMR and genomic characterization of MRSA isolated from ground pork, retail whole chicken, and patient samples in Hanzhong, China. Their data showed that 83.9% MRSA isolates expressed an MDR phenotype and three dominant livestock-associated MRSA (LA-MRSA) sequence types were identified: ST59-t437, ST9-t899, and ST398, among which, the previous major human MRSA ST59 had become the predominant interspecies MRSA sequence type among humans and retail livestock products with high adaption and transmission capacity, warranting special attention and active surveillance in China. In view of the fact that *S. aureus* has developed resistance against glycopeptides which were considered the last drug of choice against MRSA, and the emergence of vancomycin-resistant, and teicoplanin-resistant strains is globally reported, Habib et al. explored the role of the *tcaRAB* operon in *S. aureus* persister cells formation using WGS and RNA sequencing technology by studying a clinical MRSA isolate from a COVID-19 patient which showed a high level of resistance to teicoplanin, vancomycin, and methicillin. Their data revealed that Δ*tcaA* mutants resulted in a significant increase in persister cell formation in comparison to the wild type and *tcaA* might be one of the key genes that increase persister cells and glycopeptide resistance and could be a potential therapeutic target in *S. aureus*, and *tcaA* inactivation would give rise to persisters that tolerated a high concentration of glycopeptides and resuscitated the bacterial population. Their findings might begin to explain in part the mechanism of persister cell formation during host infection of MRSA.

*K. pneumoniae* has an exceptional ability to acquire exogenous resistance-encoding and hypervirulence-encoding genetic elements and the emergence of carbapenem-resistant *K. pneumoniae* (CRKP) has led to the extremely limited antibiotic therapy options (Ernst et al., 2020). At first, concurrent hypervirulence and MDR phenotypes have not been reported in *K. pneumoniae*. However, hypervirulent CRKP (hv-CRKP) isolates have been increasingly reported in recent years. The epidemic of hv-CRKP strains have emerged as a worldwide public health concern as they may cause untreatable, severe infections (Yang et al., 2021). Wu et al. reported the uncommon co-existence of *bla*_OXA − 232_, *rmtF*, and a mobile pLVPK-like virulence plasmid in a ST15 CRKP isolate, after the screening of 207 CRKP isolates from patients at 12 tertiary China teaching hospitals in eight Chinese provinces from January 2015 to May 2021. It not only exhibited resistance to carbapenems and high-level resistance to aminoglycosides, but also possessed typical pathogenic characteristic, including hypermucoviscosity and hypervirulence phenotypes. Notably, they found the co-existence of resistance and virulence plasmids not only generated the high-risk hypervirulent multidrug-resistant phenotype, but also increased the transmission of non-conjugative virulence plasmid. Jiang et al. attempted to characterize the differences in molecular characteristics and expression of virulence genes between 150 clinical isolates of CRKP and 213 isolates of carbapenem-sensitive *K. pneumoniae* (CSKP) from the local area in Ningbo, China. Their data showed that CSKP could carry a significantly greater number of virulence genes and higher virulence gene expression efficiency and virulence phenotype than CRKP. Compared to CSKP, CRKP strains had noticeable homogeneity with ST11 being the predominant sequence type, whereas CSKP strains exhibited relative diversity, but there is still some evidence of clonal dissemination (ST23).

*B. cereus* is one of the leading etiological agents of toxin-induced foodborne diseases, and its omnipresence in different environments, its capacity for spore formation, and its ability to adapt to varying conditions and produce harmful toxins make this pathogen a health hazard that should not be underestimated; food poisoning attributed to *B. cereus* can manifest itself as an emetic or diarrheal syndrome which were caused by the potent peptide toxin cereulide and proteinaceous enterotoxins, respectively (Jovanovic et al., 2021). As a foodborne pathogen, as well as a causative agent of non-gastrointestinal infections and even nosocomial complications, *B. cereus* has inspired vast volumes of multidisciplinary research in food and clinical domains. However, we only collected one manuscript in our Research Topic, Zhou Q. et al. reported nine strains of *B. cereus* from four foodborne outbreaks in Guizhou Province in southwest China collected from June to September 2021. WGS, comparative genomic and secondary metabolite analysis were performed to give a thorough exploration in terms of the mechanism of toxin production. They observed a contraction of gene families among the isolates, which were mainly associated with prophages and which contributed to the species diversity of *B. cereus*. The Hsp20 gene family underwent a rapid evolution in these strains, which facilitated the adaptation to adverse environmental conditions. They also found a higher copy number in the non-ribosomal polypeptide synthetase (NRPS) genes and found isolates carried the complete cereulide synthetase (ces) gene cluster sequences, which is a classical regulatory mechanism for emetic toxin synthesis. These findings are important for further investigation into the evolutionary relationship between *B. cereus* and their related species, as well as the underlying mechanisms governing the synthesis and secretion of bacterial toxins.

It was thought that housemaids operating inside a kitchen could be the source of infection and may transmit disease-inflicting pathogens through contaminated hands. This is an interesting hypothesis that historically was considered to be linked to cases such as Typhoid Mary in the United States. To assess the prevalence and antimicrobial susceptibility profile of bacteria isolated from the hands of housemaids in Jimma City, Ethiopia, a laboratory-based cross-sectional study including 234 housemaids were carried out by Ango et al.. The proportion of housemaids' hands containing one or more positive bacterial isolates was 72%, and the dominant bacterial isolates included *S. aureus* (31.6%), *E. coli* (21.3%), *Klebsiella* species (23.1%), *Proteus* species (14.7%), which accounts for the proportion of 90%, following by *Shigella* species (6.7%) and *Salmonella* species (1.3%). Fingernail status and the removal of a watch, ring, and bracelet during hand washing were significantly associated with the prevalence of bacterial isolation. Most of the bacterial isolates were susceptible to chloramphenicol and gentamycin, while the majority of them were resistant to tetracycline, vancomycin, and ceftazidime.

Due to the long-term and inappropriate use of antibiotics for the prevention and control of bacterial diseases in aquaculture, ARGs have become a new source of pollution in aquatic products. Factors such as the spread of drug-resistant strains and the horizontal transfer of drug-resistant genes have led to multi-drug resistance in fish pathogens, which seriously affects the quality and safety of aquatic products. Gao et al. characterized the phenotypic characteristics of the bacteria to common drugs used in aquaculture such as sulfonamides, amide alcohols, quinolones, aminoglycosides and tetracyclines, and screened for AMR genes in horse mackerel and puffer fish products sold in aquatic product markets and seafood supermarkets in Dalian, China. Their statistical analyses demonstrated that the drug resistance phenotypes and genotypes of bacteria detected in marine aquaculture fish samples in the Bohai Sea area of Dalian were complex, and the multi-drug resistance rate reached 80%, which might be closely related to the practice of intensive aquaculture. The detection rate of the AMR genes *tetA, sul1, sul2, qnrA, qnrS*, and *floR* exceeded 70% and all samples carried more than three drug resistance genes. Their data also indicated that the aminoglycosides gentamicin and tobramycin could still be considered effective in controlling bacterial infection in marine fish in the study area.

In this Research Topic, we also included a study on the analysis of clinical drug resistance mechanisms of the fungus *Aspergillus fumigatus*, and a new method of multi-detection and identification of food-borne pathogens in agricultural wastewater using next-generation sequencing technology. Zeng et al. investigated the biological characteristics of five strains of *A. fumigatus* in terms of sporulation, biofilm formation, evasion of phagocytosis, virulence, and drug sensitivity. A series of comprehensive experimental techniques, including Sanger sequencing, three-dimensional (3D) protein construction, high-performance liquid chromatography (HPLC), real-time quantitative polymerase chain reaction (RT-qPCR), biochemical analyses and transcriptomics, were employed to explore and clarify the mechanisms underlying drug resistance of the five strains. They found notable differences in the biological characteristics and pathogenic ability among the five test strains. The *cyp51A* mutations combined with the *hmg1* mutation S541G were associated with the mechanism of ITR resistance; the VRC resistance mechanism was associated with *cyp51A* mutations, changes in energy production, and increased expression of genes drug efflux pumps. Park et al. developed and optimized a novel NGS panel method that achieves the rapid and accurate detection and identification of 18 specific virulence factor genes from six target food-borne pathogens (*Bacillus cereus, Yersinia enterocolitica, Staphylococcus aureus, Vibrio cholerae, Vibrio parahaemolyticus*, and *Vibrio vulnificus*) in contaminated samples with efficiency, sensitivity, and accuracy. They introduced the term “NGS panel” to the readers, which refers to an NGS-based assay that allows for the simultaneous analysis of multiple genes, genetic variants, microbial genomes, or other genomic features, with which hundreds to thousands of target gene sequences can be screened at once and many samples can be simultaneously analyzed to rapidly and efficiently detect and identify various foodborne pathogens. It also avoids the issue of producing an overabundance of sequence information which occur in 16S rRNA sequencing based on Sanger sequencing, 16S rRNA-based metagenome and random genome sequencing-based shotgun metagenomics approaches prior to the use of NGS panels. Therefore, this technology could be useful for ensuring food safety through the prevention of foodborne disease outbreaks via the rapid and accurate detection and identification of foodborne pathogens.

In conclusion, this Research Topic provides a platform for recent discoveries and the latest progress in detection, mechanisms, and dissemination from omics insights with regards to the emerging or re-emerging foodborne pathogens with high-level AMR (Multi-drug resistant/Extensively-drug resistant/Pan-drug resistant, MDR/XDR/PDR) or hypervirulence, expending our current understanding of these superbugs, and enabling us to track their sources, to discover the mechanisms and dissemination paths along the animal-food-human chain. This Research Topic presented 15 papers that showed important information and new directions for related research, contributing to a better understanding of AMR and pathogenic mechanisms. Simultaneously, the concept of an integrated multi-disciplinary “One Health” approach has also been mentioned for widespread and sustained surveillance of foodborne pathogens, based on a multi-sectoral collaboration framework, to mitigate and prevent the threats of pathogens of animal-, human-, environment-, and food-origins (Erkyihun and Alemayehu, 2022). Moreover, this Research Topic emphasises the necessity for continued multi-disciplinary research and surveillance to mitigate the threats posed by these formidable pathogens.

Whereas, humans and pathogenic bacteria have coexisted in this earth and been intertwined through all of humanity's history. Despite recent progresses in this struggle, mankind has not really defeated these microbes, and emerging and re-emerging infectious diseases have long been a major threat to human health, since they are always on a path of mutation, development, and evolution, however, we still know very little about these aspects. For this reason, further research is needed to understand the terms of pathogenicity, the evolutionary arms race, and prevention in our food supplies. We are planning a sequel series of this Research Topic, which will aim to focus more on the virulence phenotype detection, pathogenic mechanism, fitness cost, toxicological evaluation techniques and risk assessment to human health of food-borne pathogenic microorganisms. We expect the next Research Topic would provide the research community with some of the most recent updates in the area.

## Author contributions

YH: Funding acquisition, Investigation, Resources, Writing – original draft. WW: Investigation, Resources, Writing – original draft, Writing – review & editing. SN: Investigation, Resources, Writing – original draft, Writing – review & editing. GM: Investigation, Resources, Writing – original draft, Writing – review & editing. FL: Methodology, Resources, Supervision, Writing – review & editing. SF: Methodology, Resources, Supervision, Writing – review & editing.
